# Microphysiological systems modeling acute respiratory distress syndrome that capture mechanical force-induced injury-inflammation-repair

**DOI:** 10.1063/1.5111549

**Published:** 2019-11-22

**Authors:** Hannah Viola, Jonathan Chang, Jocelyn R. Grunwell, Louise Hecker, Rabindra Tirouvanziam, James B. Grotberg, Shuichi Takayama

**Affiliations:** 1 School of Chemical and Biomolecular Engineering, Georgia Institute of Technology, Atlanta, Georgia 30332, USA; 2 The Parker H. Petit Institute for Bioengineering and Bioscience, Georgia Institute of Technology, Atlanta, Georgia 30332, USA; 3 Wallace H. Coulter Department of Biomedical Engineering, Georgia Institute of Technology and Emory School of Medicine, Atlanta, Georgia 30332, USA; 4 Department of Pediatrics, Division of Critical Care Medicine, Children's Healthcare of Atlanta at Egleston, Emory University School of Medicine, Atlanta, Georgia 30322, USA; 5 Division of Pulmonary, Allergy and Critical Care and Sleep Medicine, University of Arizona, Tucson, Arizona 85724, USA and Southern Arizona Veterans Affairs Health Care System, Tucson, Arizona 85723, USA; 6 Department of Pediatrics, Emory University School of Medicine, Atlanta, Georgia 30322, USA and Center for CF and Airways Disease Research, Children's Healthcare of Atlanta, Atlanta, Georgia 30322, USA; 7 Department of Biomedical Engineering, University of Michigan, Ann Arbor, Michigan 48109, USA

## Abstract

Complex *in vitro* models of the tissue microenvironment, termed microphysiological systems, have enormous potential to transform the process of discovering drugs and disease mechanisms. Such a paradigm shift is urgently needed in acute respiratory distress syndrome (ARDS), an acute lung condition with no successful therapies and a 40% mortality rate. Here, we consider how microphysiological systems could improve understanding of biological mechanisms driving ARDS and ultimately improve the success of therapies in clinical trials. We first discuss how microphysiological systems could explain the biological mechanisms underlying the segregation of ARDS patients into two clinically distinct phenotypes. Then, we contend that ARDS-mimetic microphysiological systems should recapitulate three critical aspects of the distal airway microenvironment, namely, mechanical force, inflammation, and fibrosis, and we review models that incorporate each of these aspects. Finally, we recognize the substantial challenges associated with combining inflammation, fibrosis, and/or mechanical force in microphysiological systems. Nevertheless, complex *in vitro* models are a novel paradigm for studying ARDS, and they could ultimately improve patient care.

## I. PATHOPHYSIOLOGY AND ENDOTYPES OF ARDS

### A. Background

Acute Respiratory Distress Syndrome (ARDS) is a life-threatening acute lung condition characterized by the sudden onset of severe pulmonary inflammation and edema resulting in secondary hypoxemia and pulmonary fibroproliferation. ARDS can be triggered by various insults, whether direct (e.g., aspiration, pneumonia, and mechanical ventilation) or indirect (e.g., sepsis, trauma, and blood transfusion).^2,8,84^ Following such insults, most ARDS patients must be placed on positive pressure mechanical ventilation that can cause ventilator-associated lung injury, which exacerbates the initial tissue injury.^101^ Despite over 50 years of intense study and attempts at pharmacological treatment, the mortality rate in ARDS patients hovers at 35%–45% and the condition afflicts an estimated 190 000 patients per year^84^ in the United States. It is also responsible for up to 10% of intensive care admissions globally.^11,35,46^ Only modest improvements in survival have been made due to mechanical ventilation strategies that minimize ventilator associated lung injury.^118^ So far, no pharmacological therapies have reduced ARDS mortality, including those aimed at attenuating inflammation, preventing or suppressing fibrosis, addressing infection, or surfactant replacement to reduce fluid mechanical stress.^46,64,67^

### B. ARDS pathophysiology

Pathophysiology of ARDS occurs in 3 chronological phases. In the exudative phase, severe inflammation causes diffuse alveolar injury and increased epithelial and microvascular permeability. Proteinaceous vascular fluid leaks into the alveolar lumen, and large amounts of alveolar epithelial cells die. Surfactant production is compromised as type II pneumocytes are lost so that the fluid flooding the lumen has an abnormally high surface tension.^28^ Leukocytes, especially neutrophils, are aggressively recruited and release proinflammatory cytokines, proteases, and neutrophil extracellular traps. Apoptotic epithelial cells and neutrophils accumulate in the alveolar lumen and begin to form hyaline membranes composed of immunoglobulin, complement, dead cell debris, and fibrin. Fibroblasts infiltrate into this environment to repair the tissue damage sustained by the initial injury. In the proliferative phase, type II alveolar epithelial cells and fibroblasts proliferate and cover sites of denudation in the alveoli. Fibroblasts become activated and deposit fibronectin to re-establish a basement membrane, and type II alveolar epithelial cells differentiate to type I epithelium and restore gas exchange and barrier function to sites of denudation. Immune cells are continuously recruited to mediate tissue repair. Hyaline membranes are a characteristic histological finding during this phase.^19^ The fibroproliferative phase is characterized by myofibroblast invasion, fibroblast proliferation, and collagen production. Surviving patients often experience a permanent decline in lung function.^22^ For detailed pathophysiology that is outside the scope of this review, readers are referred to recent comprehensive reviews by Matthay *et al.*^84^ and Sapru *et al.*^108^

### C. Injury-inflammation-repair in ARDS

**FIG. 1.**
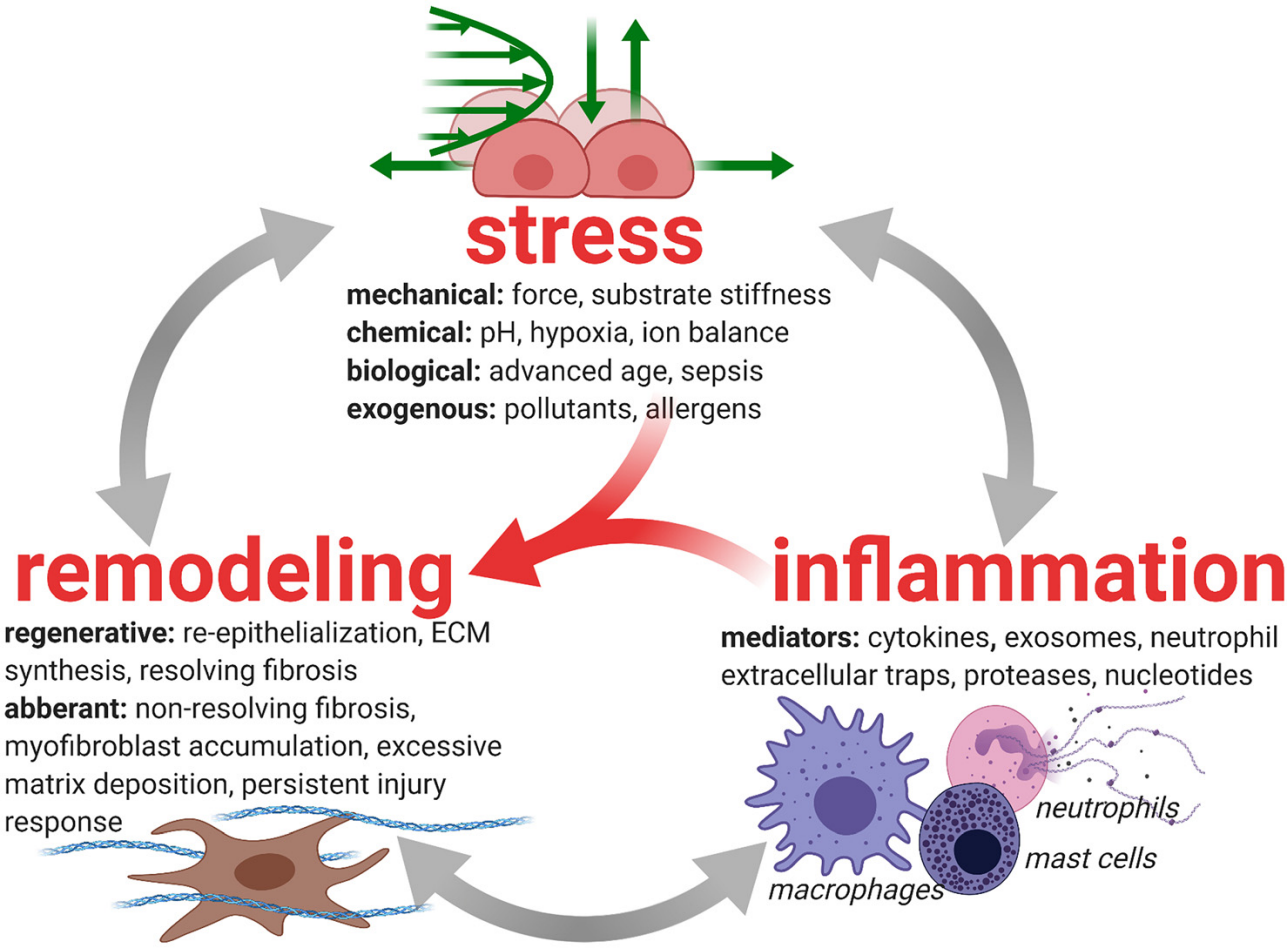
Injury-inflammation-repair in lung disease. Inflammation and fibrosis (resolving or nonresolving) cooperate to remodel lung tissue after injury.^7,27^ Both processes are heavily influenced by stressors in the tissue microenvironment. In ARDS, mechanical forces arising from surfactant dysfunction^28^ and mechanical ventilation^101^ interfere with the tissue repair process by reinjuring the tissue,^2,29,53,69^ thereby inducing inflammation^123^ and promoting fibrosis.^15,121,131^ The interactions between tissue stress, inflammation, and remodeling can direct the tissue toward successful tissue repair or toward aberrant remodeling that consists of progressive fibrosis and systemically dysregulated immunity.^7,15,88,89,131^

Tissue repair, especially restoration of barrier function, is coordinated through inflammatory and fibrotic processes that are influenced by the mechanical and biochemical microenvironment [Fig. 1 (Refs. 2, 7, 15, 27–29, 53, 69, 88, 89, 101, 121, and 123)]. The initial injury is caused primarily by neutrophil-dependent and platelet-dependent damage to the endothelial and epithelial barriers of the small airways and alveoli. Studies in large animals showed that alveolar edema occurs only when there is damage to both the endothelium and epithelium.^138^ In healthy repair, inflammation and fibrosis restore barrier and gas exchange function to the epithelium and endothelium and subsequently resolve.^4,7,12,27^ While the endothelium's morphology appears unaffected aside from its compromised barrier function, the epithelium experiences significant denudation and apoptosis in addition to loss of barrier function, which prevents removal of alveolar edema fluid and deprives the lung of adequate quantities of surfactant.^84^

**FIG. 2.**
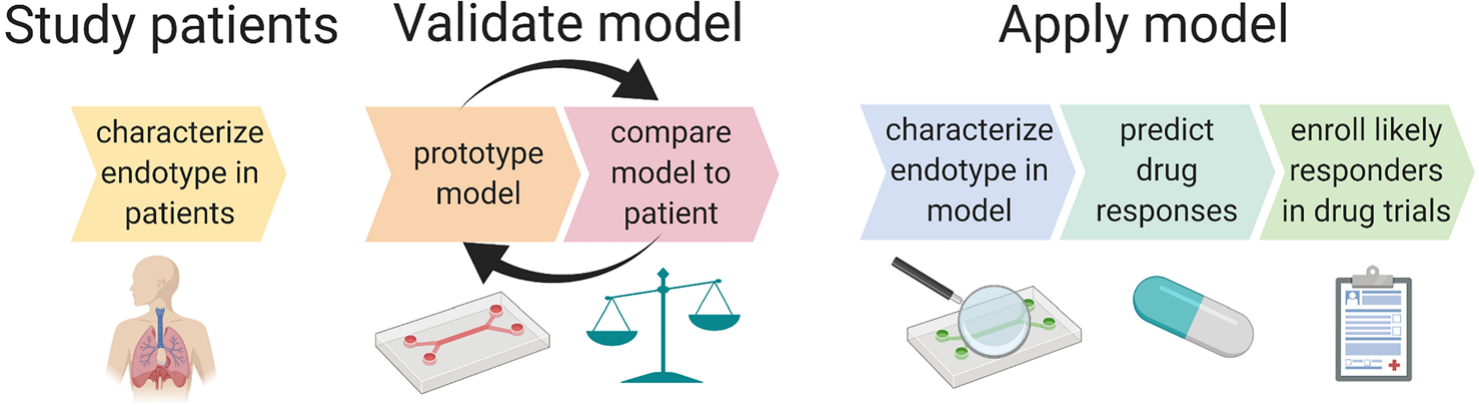
Iterative model design with validation against patient phenotype will lead to an endotype-specific model of ARDS that can be used for predictive enrichment of ARDS clinical trials. Endotype-specific metrics such as cytokine ratios, immune cell functions (e.g., bacterial killing, metabolism, and NETosis), and degree of surfactant production can be compared between patients and *in vitro* models. Iterative adjustments to model parameters, such as genetics (e.g., MUC5A upregulated epithelium), physical forces, degree of initial injury, degree of fibrosis, and the type and ratio of inflammatory mediators, could enable the development of a model that produces biomarkers or functional characteristics (e.g., response to therapeutics, response to mechanical strain, and immune cell phenotype changes such as enhanced NETosis), mimicking those of a specific endotype. The model should also be validated by testing functional outputs such as barrier function of the epithelium and tissue healing (scratch wound assay). The final model provides the opportunity for pathophysiological mechanisms of disease to be clarified and for drug candidates to be tested *in vitro*. Both pathophysiology and drug testing will help predict whether a certain endotype is likely to respond to novel treatments.

Epithelial repair in ARDS is often dysregulated. Blood neutrophils are recruited massively to the lumen, where they extend their lifespan in the lung tissue and perpetuate inflammation.^48^ Meanwhile, activated fibroblasts deposit excess collagen that impairs gas exchange.^86^ Most patients require mechanical ventilation and experience surfactant dysfunction, exacerbating epithelial injury during the repair process and imparting sublethal stresses on the epithelium. Compounding the impact of ventilation is the fact that the epithelium, immune cells, and fibroblasts sense and respond to mechanical forces^14,31,60,85,109,121,137,144^ in the lung microenvironment. Therefore, models of inflammation and fibrosis during mechanical ventilation are critical to understanding how epithelial repair impacts ARDS endotype development and consequently the patient's chance of survival.

### D. ARDS endotypes

ARDS is a clinically heterogeneous condition. Approximately 10% of ARDS patients recover rapidly and in the acute phase (<3 days),^110^ and these patients may not require intervention aside from ventilation. Meanwhile, a larger subset of patients experiences progressive fibrosis^80,81,89^ and/or systemically dysregulated inflammation,^17,90,104^ both of which are associated with a higher risk of mortality.^17,21,65,81,87,118^ These nonresolving patients might benefit from pharmacologic intervention, especially if their risk of developing to this stage could be identified prior to the onset of symptoms. Additionally, ARDS clinical trials^74,106,128^ have reported inconsistent therapeutic responses. This failure could be explained by the syndrome's heterogeneity. Therefore, there is great interest in predicting (a) which patients will not recover rapidly to determine when intensive treatment and/or trial enrollment is most beneficial^38^ and (b) which nonresolving patients might respond to which therapeutics to enrich clinical trial cohorts with potential responders.

To address these urgent questions, clinicians have developed prognostic markers that correlate ARDS outcomes with epidemiology, genomics, clinical features, physiology, and biomarkers.^26,116^ The ladder classifier is advantageous because it stratifies patients based on indicators of the underlyling biology of their disease. This powerful connection to biology might enable clinicians to predict therapeutic responses using these biomarker-based classificiations when the pathophysiology driving each biomarker profile is better understood. For example, Calfee *et al.*^17^ used latent class analysis to show that ARDS patients cluster into two clinical endotypes based on biomarkers: hyper- and hypoinflammatory. The former experienced higher rates of shock and metabolic acidosis, had significantly worse outcomes, and had higher mortality in response to mechanical ventilation with low positive end expiratory pressure. These findings were later verified using cluster analysis,^13^ and a second retrospective trial analysis showed endotype-associated responses to simvastatin.^18^

In these retrospective studies, Calfee's classifiers have demonstrated the potential to transform patient care by treating patients based on the biology driving their disease. However, biomarker-based endotyping cannot fulfill its promise of predicting endotype-specific responses to drugs until the biological mechanisms behind each endotype are understood. Only then will endotyping be a convincing determinant of patient enrollment in clinical trials. Sinha and Calfee^116^ provide a more extensive review of ARDS endotyping and the need for biological mechanisms.

## II. MODELING ARDS ENDOTYPES

### A. Traditional models

To understand the biological mechanisms that drive ARDS endotypes, preclinical models of ARDS pathophysiology are essential. The ideal preclinical model of ARDS pathophysiology should recapitulate only the critical aspects of the complex disease microenvironment, focusing on a specific etiology and patient endotype. Current models are limited in their ability to represent human pathophysiology for the study of disease and drug mechanisms.

2D monoculture of the airway epithelium *in vitro* cannot capture intricacies of inflammatory networks and cross talk between processes of injury, inflammation, and remodeling. This culture method typically also neglects tissue-level stresses such as mechanical force and does not account for fluid stresses that are dominant in ARDS due to surfactant depletion. Finally, cell lines are limited in their relevance to pathophysiology. However, primary human cells are becoming more accessible. For example, the Marisco Lung Institute's CF Center Tissue Procurement and Cell Culture Core has pioneered isolation and culture techniques for primary human lung cells.^43^

Animal models, notably mouse models, capture complex interactions between injury, inflammation, and tissue repair in ARDS, making them more suitable than current *in vitro* studies for drug testing. For pathophysiology studies, however, species-specific differences in lung physiology could interfere with attempts to correlate biomarkers with pathophysiological mechanisms. There is conflicting evidence regarding whether murine gene expression profiles in response to lung injury correlate well with those in humans. Sweeney *et al.* argue for significant similarity between murine and human inflammation after lung injury,^122^ although limitations to this study include the small human sample size (n = 3) and the number of genes evaluated (n = 432). Further, this study compared human samples from patients with non-ARDS lung injury. Seok *et al.*, in contrast, assert that when comparing almost 5000 human and murine genes altered by the same inflammatory stressors (i.e., burn, trauma, and hypoxemia), mouse models of inflammation show a close to random (R^2^ between 0 and 0.1) association to human gene counterparts.^112^ Inflammation in ARDS involves thousands of responsive genes,^104^ and a comprehensive determination has not been reached about the relevance of murine gene expression to human ARDS. Therefore, there is interest in studying human cells to complement animal studies.

Inherent limitations hinder the study of primary human samples. It is impossible to control the cell types (e.g., immune cells, epithelium, and fibroblasts) and mediators (e.g., cytokines, chemokines, and extracellular matrix components) present in a patient's lung microenvironment, limiting the ability to interrogate individual components' contribution to pathophysiology. This limitation can result in studies that are descriptive rather than mechanistic. Biopsy samples are acquired primarily from deceased patients because biopsies are a high-risk procedure for living patients. Because of this, human lung samples are biased toward severe disease and provide little opportunity to study the evolution of the disease microenvironment from the early to late stage. Bronchoalveolar lavage fluid (BALF) provides a snapshot of the distal lung's cytokine, immune cell, and mucus content, but cellular-level mechanisms cannot be positively inferred without corroborating *in vitro* data.

Whole blood is readily available but provides limited information about the lung microenvironment. Particularly, peripheral neutrophils are often studied in ARDS, but their relevance to the lung microenvironment is unclear because lung neutrophils appear to acquire novel phenotypes upon recruitment to the airways. *In vitro*, transepithelial migration of primary peripheral neutrophils into pediatric ARDS patient airway fluid activates neutrophils toward a proinflammatory phenotype with paradoxically decreased bacterial killing potency.^51^ Additionally, primary neutrophils isolated from adult ARDS BALF display impaired bacterial killing and superoxide production compared to blood and local arterial neutrophils.^83^ Overall, human samples are a vital component of ARDS pathophysiology research but corresponding *in vitro* data from more sophisticated models are needed to study mechanisms. For a more in-depth review of preclinical ARDS models, see the excellent analysis by Laffey and Kavanagh.^72^

### B. Modeling ARDS in microphysiological systems (MPSs)

Microphysiological systems (MPSs) are *in vitro* cell culture systems incorporating 3-D culture, coculture, physical forces, or other tissue-level phenomena that aim to create a tissue-mimetic microenvironment. They capture complex tissue-level physiology and disease phenomena *in vitro* using primary human cells and fluids from patients,^68^ lending them the potential to bridge the gap between animal models and human pathophysiology. MPSs supplement human and animal models and provide a third paradigm for the study of complex disease processes and drug mechanisms. MPS modeling different ARDS endotypes could explain the different responses to simvastatin and protective ventilation that were observed between hyperinflammatory and hypoinflammatory cohorts in clinical trials.

Of course, the challenge of recreating a pathophysiology that is poorly understood, for the purpose of advancing its understanding, cannot be overstated. ARDS endotypes are not correlated strongly with disease etiology or epidemiology, and as a result, there are currently no preclinical models of endotype-specific ARDS. There are several excellent etiology-specific models: Hecker and colleagues recently developed a “two-hit” murine model of ARDS combined with aging that resembles human ARDS more closely than traditional mouse models and may prove more clinically relevant for therapeutic efficacy evaluation.^99^ Nemzek *et al.* have studied a two-hit aspiration-induced ARDS model.^96^ However, these models do not capture endotype-specific ARDS.

MPSs are uniquely suited to become the first preclinical ARDS endotype models. MPSs can undergo iterative prototyping until a desired pathophysiological feature is adequately captured. This strategy is illustrated in Fig. 2. MPS designers compare their prototype to the human phenotype using metrics such as biomarkers, immune cell phenotypes, and responses to stimuli (e.g., strain, hypoxia, and infection). The prototype is then adjusted to better reflect *in vivo* metrics through the precise control of microenvironmental factors such as cell types, inflammatory and fibrotic mediators, and type/degree of mechanical force. MPSs capture disease processes to the extent necessary to produce endotype-specific biomarkers but remain simple enough to obtain a high signal-to-noise ratio, which is a desirable feature of *in vitro* models.

This review focuses on the bioengineering challenges of constructing an ARDS microphysiological system that could address clinical problems, especially the need to understand endotype pathophysiology. Critical aspects of the alveolar microenvironment during ARDS should be identified and included in experimental models of ARDS. This review highlights mechanical forces, inflammation, and fibrosis because they are central to ARDS pathophysiology and tissue repair, difficult to capture *in vitro* with traditional methods, and readily adaptable to existing MPS technology.

## III. CAPTURING MECHANICAL FORCES IN MPS

### A. Physiological forces in the distal airways

**FIG. 3.**
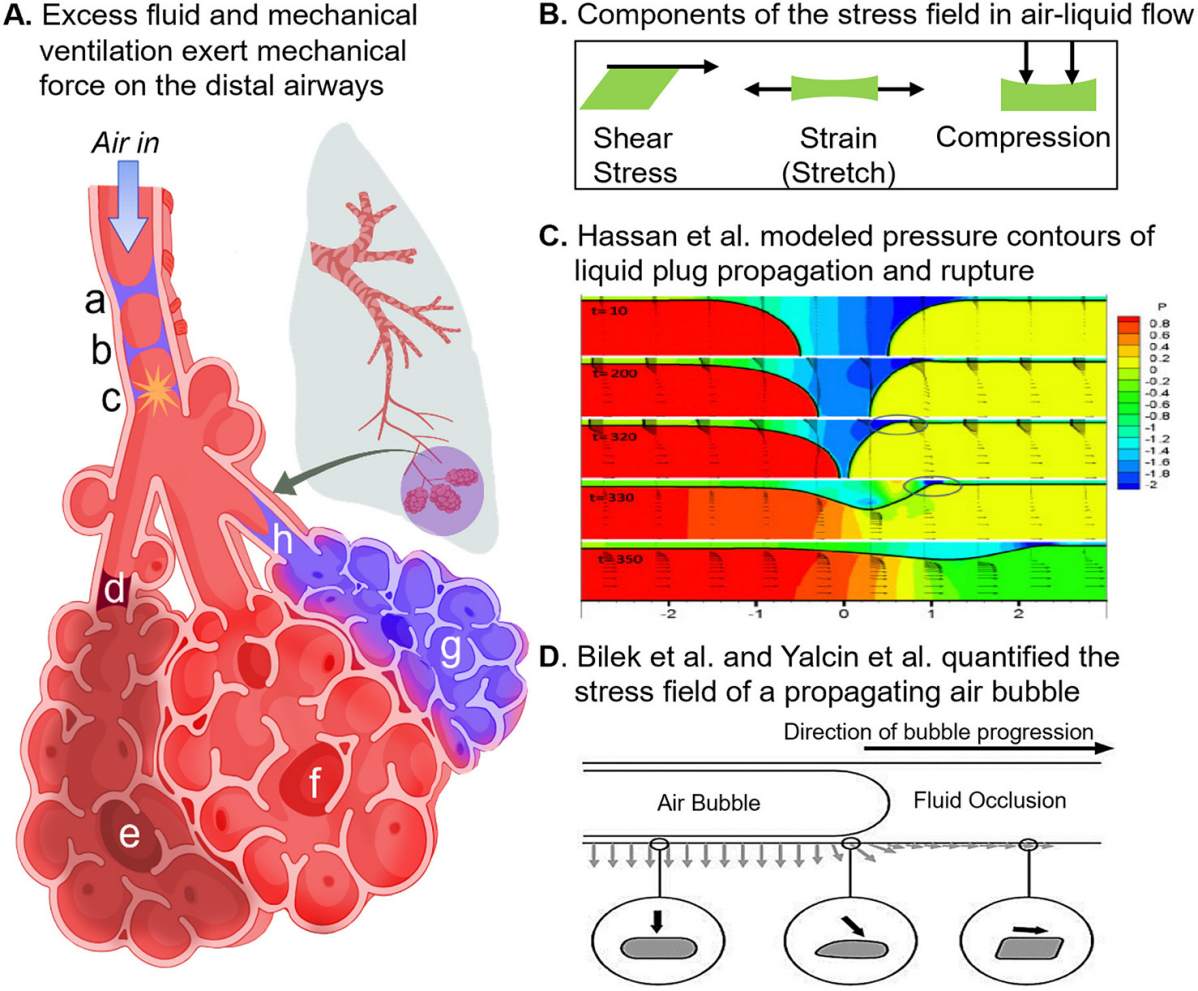
Physiologic mechanical forces in the bronchoalveolar region and their computational models. (A) The acinus consists of alveoli sacs that branch off of common terminal bronchiole (d) or (h); sacs (e), (f), and (g) are depicted in this figure. Sac (e) is cut off from air flow by the stagnant plug at (d); sac (f) is overinflated, and sac (g) is flooded with proteinaceous fluid. (B) Shear, strain, and compression are the main components of force present in the lungs, either independently or in concert. In the above depiction, strain results from overinflation of sac (f) due to obstruction of sac (d) and collapse of sac (g). Compression of adjacent sac results from the overinflation of (f). Shear stress is a component of the stress field produced during airway reopening at (h).^10,142^ Interfacial flow damages the small airways when liquid plugs propagate and rupture during inspiration^61,126^ (a)–(c) Transient liquid plugs form when the small airways collapse slightly and liquid on either side of the airways meets, forming the plug depicted in (a). Upon inspiration, the plug is pushed by pressure-driven flow, becoming thinner and thinner (b) until it loses integrity and pops (c), creating the crackle sounds that are observed upon auscultation of the lungs. (C) Hassan *et al.*^54^ modeled liquid plug propagation and rupture and found that the leading edge of the plug creates a narrow capillary wave (circled). The wave's extreme pressure gradient imparts severe stress on the airway wall. (D) The first *in vitro* model of airway reopening was introduced by Bilek *et al.*^10^ Using this model, Yalcin *et al.* found that smaller airway diameters experience greater stress.^142^ Reproduced with permission from Hassan *et al.*, Int. J. Numer. Methods Fluids **67**, 1373 (2011). Copyright 2011, John Wiley and Sons.^54^ Reproduced with permission from Bilek *et al.* J. Appl. Physiol. **94**, 770 (2003). Copyright 2003, American Physiological Society.^10^

Because a majority of ARDS patients are mechanically ventilated, it is important to understand how the forces inflicted by positive pressure ventilation and mechanical stress from excess fluid in the bronchoalveolar region impact the tissue repair process. After ARDS onset, surfactant function is compromised due to the death of type II alveolar epithelial cells and flooding of distal airways with proteinaceous fluid.^8^ This increases the surface tension of the liquid covering the surface of the alveolar and small airway epithelia, leading to abnormally high levels of fluid stresses. These are exacerbated by alveolar collapse and reopening (atelectasis), overdistension of the alveoli (volutrauma), and/or high peak inspiratory or driving pressures (barotrauma) during mechanical ventilation.^45,97,98^ Figure 3(A) illustrates forces experienced by the small airways and alveoli during ARDS. Of note, surfactant trials to alleviate fluid stress have not succeeded. Studies suggest they may have failed as a result of inadequate delivery to the alveoli due to low instilled dose volume, as indicated by computational modeling.^39,50^

### B. Existing mechanical force MPS

Lung mechanical forces can be categorized broadly as compressive stress, shear stress, and stretch [Fig. 3(B)].^140^ Shear stress is the force per area that acts parallel to a plane, often considered a “slipping” force. Strain (stretch) is the change in the length of a plane divided by the initial length. Compressive stress is the force per area applied perpendicular to a plane; it includes pressure and normal force. The effects of mechanical forces on pulmonary epithelia have been studied *in vitro* for several decades.^76,137^ Most models incorporate membranes that allow for strain or compressive stress to be applied to well-differentiated cell lines or primary airway cells in air-liquid interface (ALI) culture (Table I).

**TABLE I.** Representative sample of *in vitro* platforms that replicate mechanical forces in the lungs.

References	Mechanical Stimuli	Disease	Cell type(s)	Model type	Force application method	Key metrics in response to force
Ressler *et al.*^105^	Compression	Asthma	Rat tracheal epithelial	Transwell	Air pressurization above culture	RNA coding for Egr-1, endothelin-1, TGF-β
Swartz *et al.*^121^	Compression	Asthma	Normal human bronchial epithelial and normal human lung fibroblast (CCL-186)	Transwell epithelium culture over fibroblasts	Air pressurization above culture	Quantitative production of collagen, fibronectin
Tschumperlin *et al.*^132^	Compression	Asthma	Normal human bronchial epithelial	Transwell	Air pressurization above culture	MAP kinase and herparin-binding epidermal growth factor (HB-EGF)
Bilek *et al.*^10^	Stress field, pressure gradient	VILI	Fetal rat pulmonary epithelial (CCL-149)	Parallel plate flow chamber	Air bubble propagation	Cell death (live/dead stain)
Chu *et al.*^24^	Compression	Asthma	Normal human bronchial epithelial	Transwell	Air pressurization above culture	Quantitative expression of epidermal growth factor receptor ligands HB-EGF, epiregulin, amphiregulin, TGF-β
Tarran *et al.*^125^	Shear stress	Cystic fibrosis	Primary human epithelial	Transwell	Phasic motion of culture	Adenosine triphosphate (ATP) release, periciliary layer thickness
Choe *et al.*^23^	Compression	Asthma	Human fetal lung fibroblast (CCL-186)	Tissue-engineered human airway wall	Dynamic lateral compressive strain	Deposition of types III and IV collagen, MMPs-2 and-9
Huh *et al.*^61^	Shear stress, pressure gradient	Pulmonary edema	Primary human small airway epithelial	Microfluidic chip	Liquid plug propagation and rupture	Cell death (live/dead stain)
Yalcin *et al.*^142^	Shear stress, pressure gradient	ARDS	Fetal rat pulmonary epithelial (CCL-149)	Height adjustable parallel plate flow chamber	Air bubble propagation	Cell death (live/dead stain)
Sidhaye *et al.*^114^	Shear stress	General	Normal human bronchial epithelial	Cell culture insert	Laminar fluid flow	Paracellular permeability
Fronius *et al.*^41^	Shear stress	Cystic fibrosis	Xenopus oocyte	Xenopus oocyte	Fluid stream	Epithelial sodium channel activation
Huh *et al.*^63^	Strain	General	Human pulmonary microvascular endothelial and alveolar epithelial, peripheral neutrophil (H441, A549, E10)	Microfluidic chip	Stretching porous membrane	ICAM-1 (endothelium), ROS generation (epithelium), nanoparticle translocation
Douville *et al.*^32^	Shear stress, strain	ARDS	Human alveolar basal epithelial (A549) and primary murine alveolar epithelial	Microfluidic chip with flexible membrane	Membrane stretch and air-liquid interface oscillation	Cell death (live/dead stain)
Jacob and Gaver^69^	Stress field, pressure gradient	ARDS	Human pulmonary epithelial (H441)	Parallel plate flow chamber	Air bubble propagation	Paracellular permeability; Distribution of tight junction proteins ZO-1 and claudin 4
Stucki *et al.*^119^	Strain	General	Bronchial epithelial (16HBE14o−), primary human pulmonary alveolar epithelial, HUVEC	Microfluidic chip	Microdiaphragm stretching	Proliferation, IL-8 secretion
Manuyakorn *et al.*^79^	Strain	Asthma	Primary bronchial fibroblasts	Flexible silastic membranes	Cyclic strain	Proteoglycans, αSMA, collagens I and III, MMPs 2 & 8, IL-8
Song *et al.*^117^	Shear stress, pressure gradient	Obstructive lung diseases	Primary human small airway epithelial (CC-2547)	Microfluidic chip	Liquid plug propagation	Cell viability, NF-κB, stress fibers

**FIG. 4.**
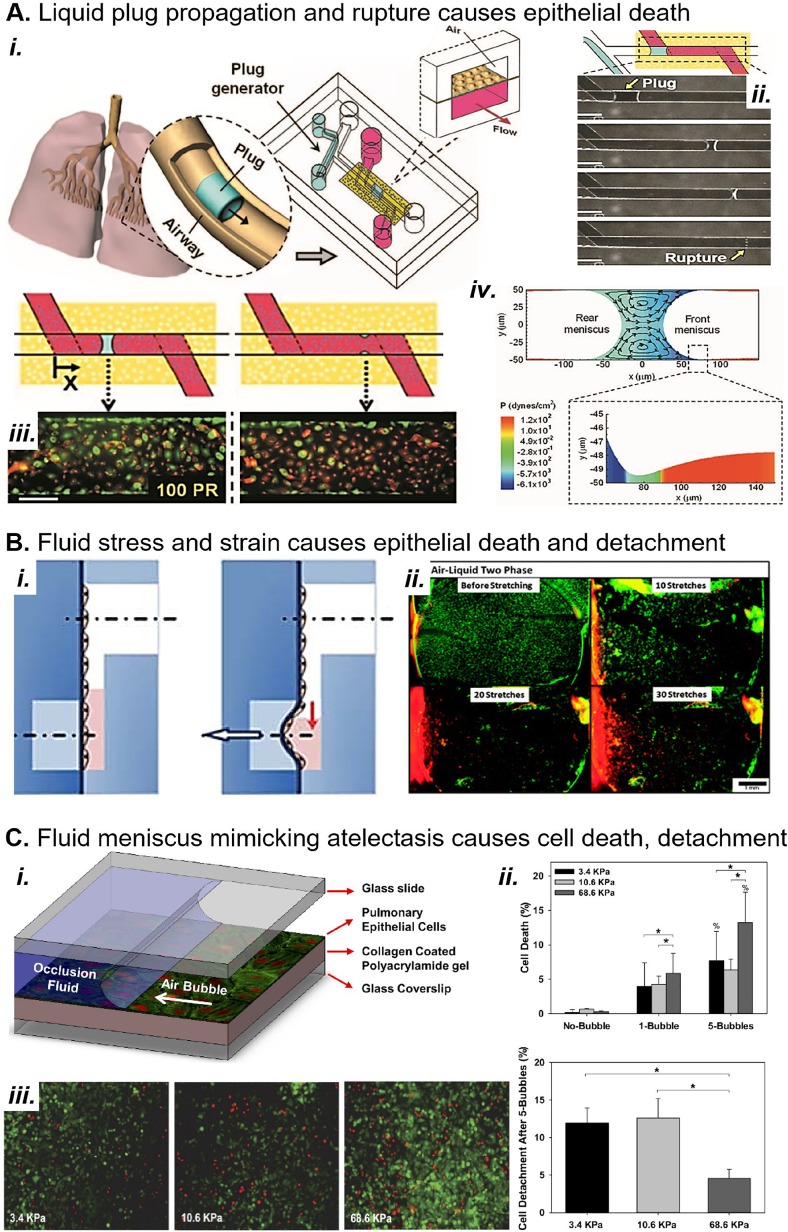
MPS models of mechanical force in lung disease. (a) (i) A microfluidic device replicates the generation of crackle sounds frequently heard in the distal airways of patients with pulmonary edema. (ii) The device generates liquid plugs that propagate and rupture in channels over alveolar type I pneumocytes. (iii) Plugs result in epithelial cell death (red) during propagation (left) and especially at the rupture site (right). Scale bar, 150 *μ*M. (iv) Fluid dynamic simulations show that the leading edge of the plug applies severe shear stress to the epithelium.^42,54^ Reproduced with permission from Huh *et al.* Proc. Natl. Acad. Sci. U. S. A. **104**(48), 18886–18891 (2007). Copyright 2007 National Academy of Sciences.^61^ (b) (i) Douville *et al.* report a device that applies simultaneous fluid shear stress and mechanical strain to alveolar epithelium; vacuum stretches the membrane and simultaneously lowers the fluid level. (ii) Fluid stress and strain result in death (red cells) and detachment of alveolar epithelium in the device. Scale bar, 1 mm. Reproduced with permission from Lab on a Chip 11, 609–619 (2011). Copyright 2011 Royal Society of Chemistry.^32^ (c) (i) Higuita-Castro *et al.* mimic small airway or alveolar reopening by propagating an air bubble over the epithelium.^57^ The device design, first conceived by Bilek *et al.*,^10^ has been extensively used to characterize the damage of liquid stress during atelectasis and airway reopening.^57,58,69,70,142^ (ii) and (iii) Higuita-Castro *et al.* show that the fluid meniscus causes increasing cell death and detachment with increasing substrate stiffness. Reproduced with permission from Higuita-Castro *et al.*, J. Appl. Physiol. **117**, 1231 (2014). Copyright 2014 American Physiological Society.^57^

Complex fluid stresses also contribute significantly to lung injury.^2,49,101,107^ Investigators have modeled the fluid stresses imparted by liquid plug propagation and rupture, small airway collapse and reopening, and alveolar collapse and reopening. The Gaver group was the first to report an *in vitro* system for modeling the stress field associated with alveolar recruitment using a moving air bubble [Fig. 3(D) (Refs. 10 and 142)]. They showed that slower bubble speeds increase cell death, despite a milder shear gradient, because the pressure gradient is significantly increased.^70^ In the same moving air bubble model, Higuita-Castro *et al.* showed that increasing the substrate stiffness caused greater cell death after 1 and 5 bubbles^57^ [Fig. 4(c)].

Additionally, Takayama and colleagues modeled liquid plug propagation and rupture *in vitro*^61,126^ [Fig. 4(a) (Refs. 42, 54, and 61)]. They found that liquid plug propagation caused cell death even without plug rupture. To explain this observation, Fujioka *et al.* showed that the front meniscus of a moving liquid plug imparts large stresses on the airway wall due to a narrow capillary wave that appears ahead of the plug's leading edge [Fig. 3(C), capillary wave circled].^42,54^ Recently, Muradoglu *et al.* also showed that surfactant reduces the mechanical stress imparted by the liquid plug.^95^ In an alveolar closure/reopening model, Douville *et al.* showed that repeat strain combined with fluid stress caused cell death and detachment, suggesting a mechanism for how atelectasis affects lung function^32^ [Fig. 4(b)]. The work of the preceding investigators has brought attention to the major role of fluid stresses in promoting lung injury. Additionally, we provide a representative history of all types of pulmonary epithelial force models in Table I.^10,23,24,32,41,61,63,69,79,105,114,117,119,121,125,132,142^

### C. Limitations of mechanical force MPS

Few pulmonary force models consider the physical properties of the extracellular matrix, such as stiffness and substrate ligands. It is well established that substrate ligands affect epithelial and endothelial properties and that focal adhesions mediate mechanosensing.^1,73^ Of relevance to mechanical force models, aligned collagen fibers in the substrate amplify cell-cell mechanotransduction across distances greater than several cell diameters.^78^ In the moving air-finger model [Fig. 4(c) (Refs. 10, 57, 58, 69, 70, and 142)], Higuita-Castro *et al.* showed that increased substrate compliance leads to greater cell detachment and less necrosis.^57^ More investigation is needed to determine the effects of substrate properties on physiology in mechanical force models.

Mechanical force models rarely contain multiple stress types, despite indications that stressors work synergistically to promote injury. For example, simultaneous surfactant loss and overstretching of the alveoli cooperatively promote secondary lung injury during mechanical ventilation.^97,98^ Even models that do capture complex force combinations or stress fields often fall short of describing the force's downstream effects on the tissue. Huh *et al.* and Douville *et al.*, shown in Figs. 4(a) and 4(b), capture complex forces but characterize only cell death and detachment. Ghadiali and colleagues, in contrast, have characterized mechanotransduction in the moving air-finger model [Fig. 4(c)].^57,58,69^ Still, both models lack immune and fibroblast coculture.

Because tissue repair is an essential component of recovery from ARDS, it is critical to capture the interaction between tissue repair and mechanical forces. This dynamic relationship remains poorly understood despite two decades of *in vitro* studies on the effects of mechanical forces on isolated epithelial, fibroblast, and immune cells.^85,127,137,144^ In the following, we discuss the challenges and opportunities for modeling this aspect of ARDS in MPS.

## IV. CAPTURING INFLAMMATION AND FIBROSIS IN MPS

### A. Inflammation and fibrosis in ARDS

Inflammation in ARDS is characterized by early and persistent neutrophilia, mast cell recruitment and activation,^135^ and recruitment of monocytes and macrophages in the distal airways and alveoli.^84^ Neutrophil activity is believed to damage tissue in ARDS.^48,146^ Neutrophils secrete proteases, such as neutrophil elastase and matrix metalloproteases (MMPs), which degrade the extracellular matrix, and neutrophils promote hypoxia through rapid oxygen consumption and the excessive production of reactive oxygen species (ROSs).^25,48,146^ Neutrophils are also affected by mechanical stressors *in vitro* and *in vivo*. Force induces the production of chemotactic factors, including TNF-α,^60,102,103,129^ IL-8,^102,131,136^ and IL-6.^103,123,129,141^ IL-6 has long been considered a biomarker of ventilator-induced lung injury, and TNF-α and IL-8 are master regulators of inflammation.

Concurrently, fibroproliferation and tissue repair proceed in response to both the primary tissue injury and the damage caused by recruited neutrophils. In the terminal bronchioles and alveoli, type II pneumocytes proliferate and fibroblasts become activated. Proliferating fibroblasts deposit provisional extracellular matrix (ECM) consisting of fibronectin, and later collagen, to repair ECM damage and restore epithelial integrity.^84,89^ Dysregulated fibroproliferative repair can develop into nonresolving fibrosis that has a poor prognosis in ARDS and is refractory to treatment.^80,81,87^ Aside from the introduction of protective ventilation strategies that limit ventilator-associated lung injury, attempts to prevent the occurrence of nonresolving fibrosis and dysregulated immunity, or to slow progression, have not yielded success.

**FIG. 5.**
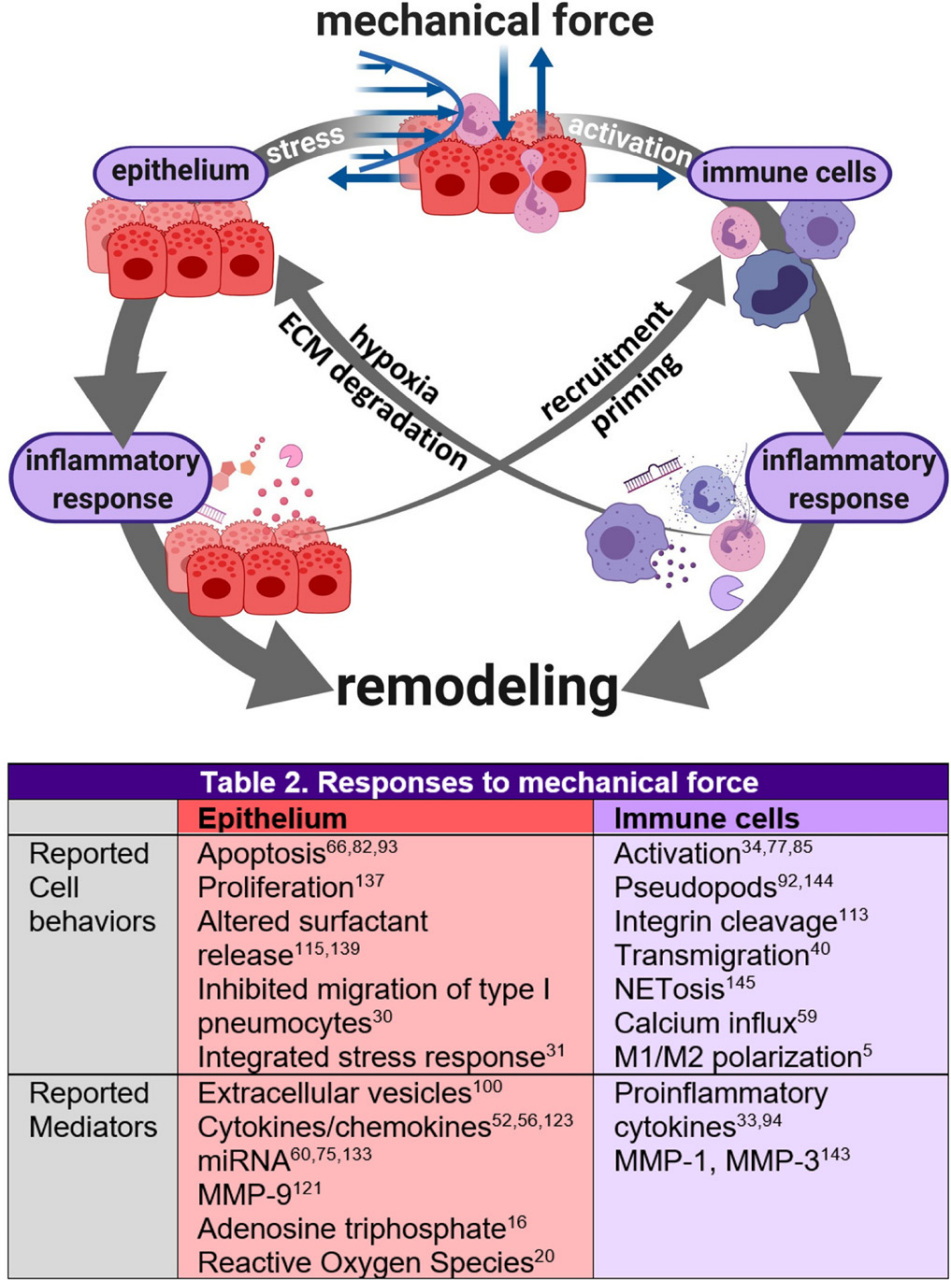
Reported mechanical stress-induced cell behaviors and inflammatory mediators that enable cross talk between pulmonary epithelium and immune cells. Mechanical stresses due to surfactant dysfunction, edema, and mechanical ventilation cause the epithelium to produce inflammatory and fibrotic mediators^107,130^ and may induce the integrated stress response, a key mediator of mechanical stress-induced epithelial injury.^31^ Mechanical force-induced cell behaviors and mediators of cross talk between the epithelium and immune cells are listed in Fig. 5. Such cross talk drives remodeling pathways; for example, hyperactivated neutrophils deplete oxygen in their microenvironment through the excessive production of reactive oxygen species (ROSs). This hypoxia stresses the epithelium and leads to apoptosis or inflammatory signaling that promotes fibroblast activation and epithelial proliferation. Neutrophils also contribute directly to remodeling during epithelial transmigration;^12^ massive neutrophil influx compromises tight junction integrity and stimulates repair of the lamina propria.^146^ Mechanically activated neutrophils and alveolar macrophages also secrete proteases that degrade the extracellular matrix (ECM), such as neutrophil elastase, and promote continued ECM destruction and activation of fibroblasts to repair the ECM damage.^27,44,124^ Because neutrophils are significant drivers of this destructive remodeling cycle, they are a target of therapeutic research.^120^

Crosstalk between the epithelium and immune system in response to mechanical force plays a significant role in mediating tissue repair. This process is poorly characterized due to its complexity and heterogeneity, but general mechanisms of mechanical force-induced epithelial-immune cross talk that have been reported individually are presented in Fig. 5 (Refs. 5, 12, 16, 20, 27, 30, 31, 33, 34, 40, 44, 48, 52, 56, 59, 60, 66, 75, 77, 82, 85, 92–94, 100, 107, 113, 115, 117, 120, 121, 123, 124, 130, 133, 137, 139, and 143–146).

### B. Challenges of modeling inflammation and fibrosis in MPS

The choice of cell types, source, and degree of activation or polarization can greatly affect cell phenotype and responses to stimuli. Tissue repair *in vivo* involves the migration, activation, and cross-contact of multiple cell types including fibroblasts, neutrophils, monocytes, and lymphocytes. The ideal model would capture a high degree of *in vivo* cell functionality including fibroblast proliferation, immune transmigration and *in situ* activation, degranulation, neutrophil extracellular trap (NET) formation, and elevated phagocytosis. However, many of these behaviors are difficult to control and modulate in complex model systems with multiple cell types. For example, TGF-β activates fibroblasts but may have concurrent effects on the epithelium and immune cells. MPSs should capture the minimal activation necessary to recapitulate the disease mechanism of interest.

**FIG. 6.**
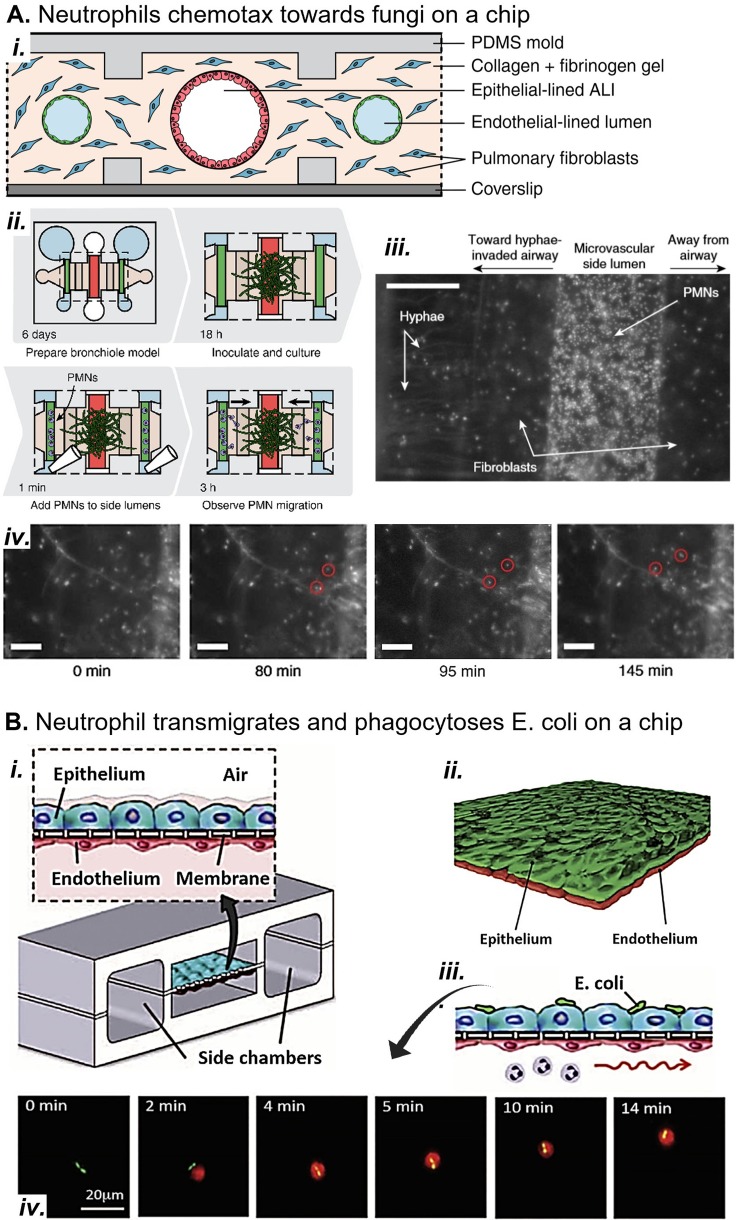
Models of pulmonary inflammation *in vitro*. (a) (i) A microfluidic small airway-on-a-chip replicates the endothelium, interstitial fibroblasts, and epithelium. **(**ii**)** Fungal infection is simulated by inoculating the epithelium with wild type *Aspergillus fumigatus*, a model fungal pathogen. **(**iii**)** Neutrophils are added to the endothelial channels after the hyphae have extended into the interstitial space. Scale bar, 200 *μ*M. (iv) Neutrophils chemotax from the endothelium through the interstitium toward fungal hyphae, attracted by volatile compounds produced by the fungus. Scale bars, 100 *μ*M. Reproduced with permission from Barkal *et al.* Nat. Commun. **8**, 1770 (2017). Copyright 2017 Authors, licensed under a CC BY 4.0 (https://creativecommons.org/licenses/by/4.0/).^6^ (b) (i) and (ii) A microfluidic alveolus-on-a-chip incorporates strain and neutrophil transmigration in a bilayer epithelium-endothelium coculture model. (iii) *E. coli* on the epithelium attracts neutrophils to transmigrate from the basal channel through endothelium and epithelium. (iv) Two *E. coli* bacteria (green) on the epithelium are chased and phagocytosed by a neutrophil (red) on the epithelium of the device. Reproduced with permission from Huh *et al.*, Science **328**, 1662 (2010). Copyright 2010 AAAS.^63^

Barkal *et al.*, for example, focused on neutrophil activation in their small airway-on-a-chip model of fungal infection. Neutrophils migrated toward volatile fungal chemoattractants through the endothelium and epithelium [Fig. 6(a)].^6^ In a model of invasive aspergillosis, the device recapitulated the well-characterized inflammatory cytokine response and increased recruitment of neutrophils observed in murine and zebrafish models. In another example, Choe *et al.* focused on activation of fibroblasts.^23^ They applied strain to a coculture of human bronchial epithelial cells atop a fibroblast-seeded collagen gel. They found that strain induced myofibroblast differentiation and type III and IV collagen deposition. Both myofibroblasts and type III collagen were concentrated close to the basal side of the epithelium, suggesting that the epithelium is a source of profibrotic mediators that promote matrix remodeling. Their *in vitro* model included the minimum cell types and activating stimuli to capture remodeling events. Furthermore, the model showed that the pathway is not mediated by immune cells because they were not present. The rational choice of the required cell types and activating stimuli enables MPSs to remain simple enough for analysis but sophisticated enough to capture inflammation and remodeling *in vitro*.

Immune cell and fibroblast cocultures greatly improve the physiological relevance of ARDS MPSs but present significant design challenges. First, very few MPSs have studied the impact of mechanical strain on fibrosis. Swartz *et al.*^121^ and Choe *et al.*^23^ showed that strain-induced fibrosis can be mediated by the epithelium, but such mechanical force pathways that induce fibrosis are likely complex and multifactorial. As such, they should be further explored in ARDS MPSs that can incorporate physiological forces in coculture systems. Additionally, the presence of multiple cell types obfuscates the source of inflammatory and fibrotic mediators (e.g., cytokines, proteases, miRNA, reactive oxygen species, and TGF-β). To overcome this hurdle, MPS data are sometimes analyzed with systems biology techniques similar to those used to parse out *in vivo* signaling pathways.^9,134^

Additionally, airway epithelia, especially from primary cells, require many days or weeks to polarize. Media optimization may not be adequate to maintain the health and desired phenotype of all cell types present in coculture in the long term. Sellgren *et al.* reported a triple coculture of primary airway epithelium, fibroblasts, and endothelium but noted that an airwaylike phenotype (cobblestone morphology, mucus production, and cilia) was difficult to maintain in coculture conditions.^111^ MPS designers should consider if long-term coculture can be avoided, and if not, what media formulations can maintain cocultured cells in their desired phenotypes.

Substrate properties and mechanical forces also affect immune and fibroblast cell phenotypes. MPSs should have physiologically relevant physical forces and substrate properties so that immune and fibroblast phenotype mimic those *in vivo*. While models have independently considered immune cell and fibroblast mechanobiology in response to single stresses such as substrate stiffness or mechanical force, few combine multiple stress types in the same microenvironment. Although Huh *et al.* (2010) [Fig. 6(b)] incorporated interstitial flow, strain, and transmigration into their alveolus on-a-chip, they did not study how these forces affected the neutrophils in the model.

## V. GENERAL CHALLENGES OF MODELING ARDS IN MPS

### A. Complexity

A major challenge of designing MPSs is determining the level of model complexity. An overly complex model will produce noisy data, but an overly simplistic one is not useful. One option is to utilize functional readouts that are already familiar to the biology community, such as phenotypic assays (e.g., assays for bacterial phagocytosis and killing by neutrophils), to reduce the dimensionality of the data while keeping the model relatively complex. MPSs are, however, limited in how complex they can become before losing physiological relevance. A model that is too complicated could create conditions that induce nonphysiological cell behaviors. Additionally, elaborate models are difficult to fabricate, which limits their throughput and accessibility to the greater research community. Designers must consider what aspect of pathophysiology they desire to model and carefully consider what features are necessary to capture the phenomenon while minimizing the components of the system.

**FIG. 7.**
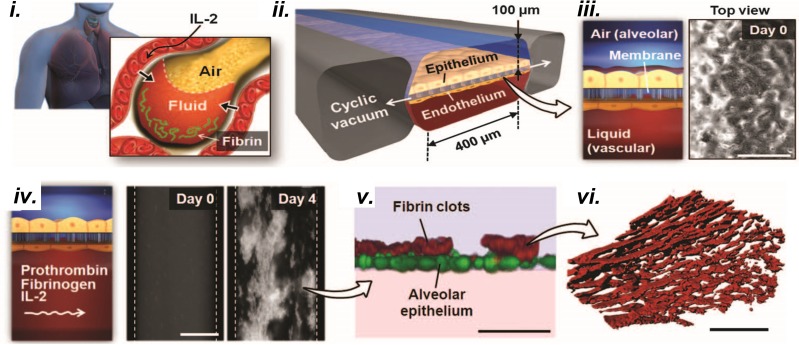
*In vitro* models of the lung microenvironment could be applied to study fibroproliferative disease in ARDS. (i) A lung-on-a-chip that replicates vascular leakage, leading to pulmonary edema and fibrin clotting.^62^ (ii) Strain is applied, by pulling vacuum on either side of the chamber, to a membrane (iii) with alveolar epithelium on the apical side and endothelium on the basal side. Scale bar, 200 *μ*M. (iv) IL-2 induces endothelial and epithelial permeability allowing basal media loaded with prothrombin and fibrin to pass through the membrane and flood the apical channel, simulating pulmonary edema. Scale bar, 200 *μ*M. (v) and (vi) Fibrin clots form on the apical channel after it becomes flooded with basal media containing fibrin and prothrombin. Scale bar (v), 50 *μ*M. Scale bar (vi), 5 *μ*M. Reproduced with permission from Huh *et al.* Sci. Transl. Med. **4**, 159ra147 (2012). Copyright 2012 AAAS.^62^

ARDS pathophysiology is complex and involves multiple stages with different characteristics. The designer must consider what aspects of disease progression to model. For example, Huh *et al.* captured pulmonary edema, fibrin deposition, and impaired gas exchange in response to toxic levels of IL-2 in a lung-on-a-chip microdevice including only the epithelium and endothelium **(**Fig. 7). They discovered that immune cells and fibroblasts were not necessary to produce these tissue-level functions, but strain was necessary, indicating that strain is a significant initiator of early pulmonary drug toxicity responses *in vivo*.

Conversely, designers must consider whether even the most complex MPS is comprehensive enough to replicate the phenomenon of interest. For example, a single MPS could not capture multiple organ failure. Systemic dysregulated immunity that is observed in sepsis is likewise unlikely to be captured in a single MPS. Many MPSs also lack an immune component, a challenge that has not been addressed sufficiently. However, the simplicity of MPSs compared to *in vivo* models is often a benefit because it allows the isolation of confounding factors from the system, such as in Choe *et al.*^23^ and Huh *et al.*^62^

### B. Heterogeneity

Because ARDS is a heterogeneous syndrome, it is impossible to construct a unifying model that incorporates every possible phenotype. MPSs could, however, be used to generate high throughput microenvironments mimicking the phenotypes to better understand divergent biological pathways driving phenotypic differences. For cell culture, primary human cell heterogeneity is also a significant challenge. Quality control of primary-cell-sourced cultures is difficult, especially in microfluidic culture with very small cell populations, due to variability across patients and even among cells from a single source. Conversely, models constructed with cell lines typically lack adequate cell heterogeneity. For example, models of the small airways that use H441 club cell lines lack the small populations of goblet cells, basal cells, and macrophages also present in this microenvironment. Most MPSs mimicking the alveoli only include alveolar type I pneumocytes and neglect type II pneumocytes, macrophages, and fibroblasts. Mertz *et al.*^91^ provide a discussion of the considerations of cell heterogeneity in MPSs.

### C. Data collection in microfluidic systems

Traditional assays are difficult to adapt to microfluidic MPSs. Epithelial barrier permeability measurement is usually absent from microfluidic devices, especially real time permeability.^55^ This measure of epithelial response to stress and recovery from injury would greatly increase the information provided by microfluidic MPSs. Similarly, the scratch wound assay is a common metric of epithelial repair and recovery from injury that has only been adapted to microfluidics by Felder *et al.*^36,37^ in a custom device. Cytokine levels produced by very small cell numbers could fall below the detection limit of Luminex or ELISA assays. The MPS designer who considers microfluidics should determine whether their study will be sensitive to these limitations.

### D. Clinical relevance

Finally, for MPSs to move from proof-of-concept to clinical applications, close cooperation with clinicians and the medical research community is essential. Clinicians connect researchers with urgent medical needs of patients and help researchers design their models in the context of a specific motivating question. Researchers in disease-specific fields provide essential information from studies of primary samples and basic science experiments that direct the design of more complex systems. An accurate model will be validated against clinical data and will recapitulate relevant aspects of ARDS pathophysiology or treatment.

## VI. OUTLOOK

Despite the challenges of using MPSs for ARDS research, opportunities abound. These models could elucidate mechanisms that drive tissue repair toward regenerative or maladaptive responses to injury in ARDS. Additionally, MPSs can be applied to study pulmonary drug delivery for surfactant replacement or other therapies.^47^ MPSs are also applicable to other lung diseases: asthma and bronchiectasis endotypes have been described recently, and similar to ARDS, little is known about the biological mechanisms behind them.^3,71^ However, both diseases also involve inflammation, remodeling, and mechanical force in the lungs.

In conclusion, ARDS is a heterogeneous syndrome with high mortality and few effective treatment options. In-depth analyses have identified subgroups of patients that respond differently to supportive interventions and have different morbidity and mortality rates, but the biological mechanisms driving these differences in outcome are unclear, hindering the translation of these phenotyping methods to patients. MPSs have transformed *in vitro* cell culture and opened the door to complex *in vitro* analysis that could uncover these biological mechanisms and accelerate the translation of new phenotyping methods to critically ill patients. Overall, MPSs have tremendous potential to reveal patient-specific biological endotypes, which would improve personalized outcomes of importance to patients.
